# The Influence of Dimensions and Powder Recycling on the Roughness and Mechanical Properties of Ti-6Al-4V Parts Fabricated by Laser Powder Bed Fusion

**DOI:** 10.3390/ma15165787

**Published:** 2022-08-22

**Authors:** Alejandro Yánez, María Paula Fiorucci, Oscar Martel, Alberto Cuadrado

**Affiliations:** Department of Mechanical Engineering, University of Las Palmas de Gran Canaria, 35001 Las Palmas de Gran Canaria, Spain

**Keywords:** laser powder bed fusion, powder recycling, titanium alloys, surface roughness, fatigue behavior

## Abstract

Powder bed fusion technology has undergone a remarkable amount of development in recent years in the field of medical implants due to the advantages associated with it. In many implant applications that demand loads in parts with a high degree of roughness and small dimensions, the mechanical properties, especially fatigue properties, play a key role in the success of the implants. One of the most used materials in this field is Ti-6Al-4V. On the other hand, the high cost of titanium powders makes it necessary to search for suitable powder recycling strategies. In this work, the effects of dimensions and powder recycling on the roughness and the mechanical properties of cylinder specimens were obtained from tensile static and fatigue tests of Ti-6Al4V Extra-Low Interstitial (ELI) parts. Four types of specimens were fabricated by laser powder bed fusion (two dimensions (section diameters of 2 mm and 5 mm) with new powder and with recycled powder). Results show that the oxygen concentration increased with recycling. No significant effects of recycling were observed on the monotonic tensile strength specimens. However, specimens fabricated with recycled powder showed greater roughness, lower ductility, and lower fatigue strength than those fabricated with new powder. On the other hand, the 5-mm-diameter specimens showed slightly better fatigue behavior than the 2-mm-diameter ones.

## 1. Introduction

Over the last several years, additive manufacturing (AM) has expanded into the industrial sector, and the most widely used material in AM has so far been the titanium alloy Ti-6Al-4V, especially for medical implants [1,2]. Besides its optimal mechanical properties, Ti-6Al-4V shows good biocompatibility and suitable osseointegration properties [3]. For this alloy, the most commonly used techniques are those based on powder bed fusion (PBF), i.e., laser powder bed fusion (LPBF) and electron beam powder bed fusion (EPBF), also known as electron beam melting (EBM), for which the heat sources are a laser and an electron beam, respectively. One of the problems that these techniques pose is that only a small percentage of the total volume of Ti-6Al-4V powder is used in each manufacturing batch. Without a proper powder recycling strategy, part manufacturing costs can become substantially higher due to the high costs of virgin metal powders [4,5]. Fortunately, Ti-6Al-4V powder can be recycled for subsequent manufacturing runs, which could lead to a reduction in manufacturing costs. On the other hand, according to both manufacturers and various studies in this regard, there are multiple powder recycling strategies. Therefore, powder recycling should be closely controlled and studied because excessive reuse could imply changes in the morphology and in the chemical composition of the powder and, consequently, alterations in the mechanical properties of the obtained parts.

Throughout the manufacturing cycle, the quality of the recycled powder should remain as close as possible to that of the virgin powder to guarantee the quality of the components. However, this is difficult to achieve because the powder degrades physically and chemically after several stages of recycling [6]. Moreover, the properties of the powder can change over time due to repeated exposure to the conditions of the manufacturing chamber and the techniques used to recycle, store, and handle it [7]. There are two common reuse strategies within the LPBF fabrication process: single-batch and top-up [6,8]. In the single-batch strategy, the powder is continually recycled, build after build, until it no longer produces parts to specifications; the unused powder is put through a sieve and then recycled until there is insufficient material left to complete a build or until the batch is unusable. In the top-up strategy, after the first batch fabrication, the unused powder is run through a sieve and then mixed with new powder to replace the material that was lost during the initial build. This process is repeated until the features of the mixture of new powder and recycled powder fall below the specified manufacturing requirements. This is repeated until the quantity of new powder available for replenishment along with the recycled powder has been depleted to below the fabrication-specified requirement.

The chemical composition of the powder represents a major parameter since it directly influences the mechanical properties of the manufactured parts [9]. The high reactivity of titanium to oxygen, particularly at high temperatures, leads to the production of Ti-6Al-4V alloys under strict control of the oxygen content because, even in small amounts, the interstitial element can change the mechanical properties markedly. Oxygen imparts strength at the expense of ductility [10,11,12]. Notably, reduced oxygen and nitrogen contents promote ductility and better fatigue behavior [10].

The literature has reported that oxygen is the element that increases the most with the successive reuse of the Ti-6Al-4V powder during PBF manufacturing. This increase is especially important when using the EBM technique since the powder oxygen concentration could be above the standard specification after a few uses [13,14,15]. This is probably because, in EBM, the pre-heating temperature is greater than that in LPBF. However, the increase in the oxygen content of the recycled Ti-6Al-4V powder fabricated by the LPBF technique has been studied only to a limited extent. It has been reported that with the LPBF technique and using the single-batch strategy, the oxygen content increased while other minor components, such as H or N, remained within the recommended limits [16].

The morphology of the metal powder also influences the quality and performance of the manufactured parts. The size of the powder particles determines the minimum thickness of the layer capable of being manufactured and the roughness of the parts. On the other hand, the particles must be as spherical as possible to achieve a homogeneous distribution in the powder bed [7]. Some authors have shown that with the reuse of titanium powder in LPBF technology, the particle size distribution (PSD) narrowed and the flowability improved [17]. However, other authors maintain that the flowability improves, but no significant changes in the PSD have been observed [6,8].

Key features to take into account in medical implants fabricated by AM are their static mechanical properties and fatigue behavior. It has been found that PBF fabrication produces parts that deviate from their original form [18]. A high degree of roughness could be an advantage for cell adhesion, but it is also a disadvantage for fatigue behavior, especially in low-thickness parts [19,20,21]. These properties may change in parts fabricated by PBF techniques with recycled Ti-6Al-4V powder. Some authors showed that with the reuse of titanium powder, no or minimal changes in the ultimate tensile strength (UTS), yield strength (YS), and Young’s Modulus (E) of parts obtained by PBF were observed, but there was an appreciable decrease in the elongation and ductility [9,15,22]. Very few works have studied the roughness and the fatigue behavior of parts obtained by PBF with recycled Ti-6Al-4V powder. Popov et al. [9] revealed a negative effect on the fatigue behavior of Ti-6Al-4V specimens fabricated by EBM. They showed a significantly shorter fatigue lifetime in specimens obtained from recycled powder. Carrion et al. [17] revealed positive effects of powder recycling on the fatigue life performance of machined Ti-6Al-4V specimens fabricated by LPBF. However, their study showed that powder recycling did not have any significant negative effects on the fatigue behavior of the Ti-6Al-4V specimens in as-built conditions.

In the present study, the roughness and the (static and fatigue) mechanical properties of specimens with sections of two different dimensions, fabricated by LPBF with new and recycled Ti-6Al-4V powder, were investigated. The main goal was to analyze how powder recycling and the different dimensions of the specimens have an impact on the properties described above. Furthermore, a fractographic analysis of the specimens after fatigue tests was carried out.

## 2. Materials and Methods

Two types of specimens (with CAD diameters of 2 mm and 5 mm) were fabricated by LPBF. Spherical Ti-6Al-4V ELI powder with a particle diameter in the range of 15–45 μm was used as the raw material, which is the most used material in medical applications. A RenAM500S (Renishaw) system was used to additively manufacture the samples. The main fabrication parameters were a 500 W laser and a 60 µm layer thickness. The fabrication parameters were recommended and certified by the manufacturer for Ti-6Al-4V. The specimens were fabricated with their tensile axis aligned with the building direction.

**Figure 1:**
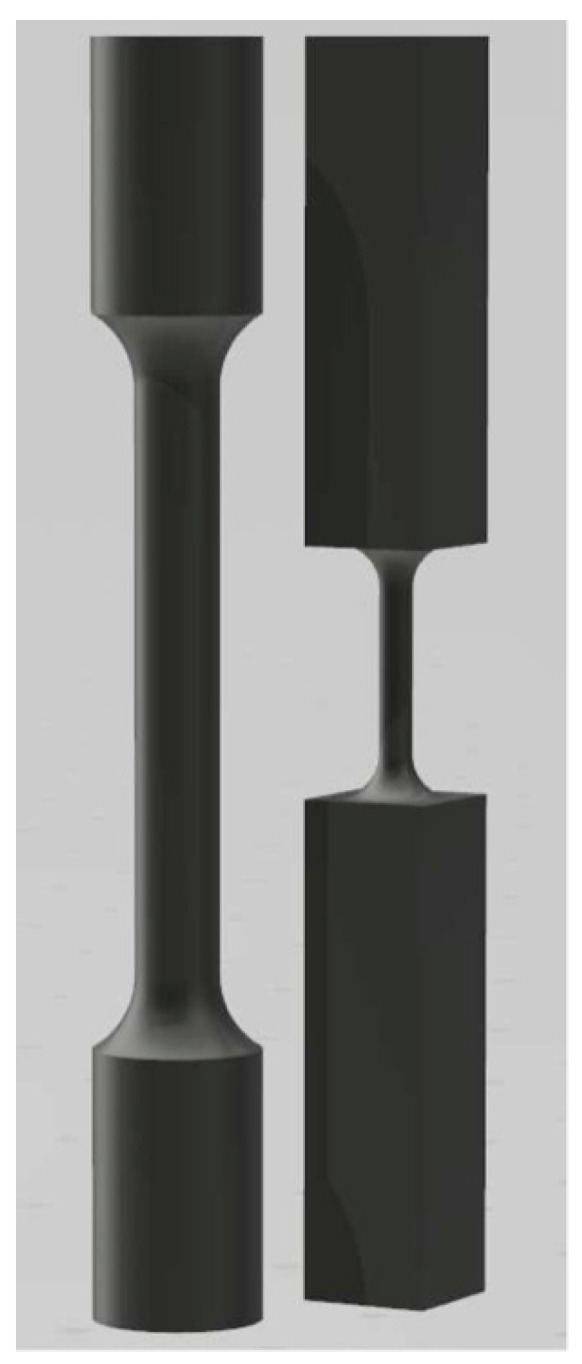
LPBF Ti-6Al-4V specimens: large diameter (**left**); small diameter (**right**).

A top-up recycling strategy was used (the used powder was replenished with new powder). After the batch was used, the titanium powder was run through a sieve and then mixed with new powder to replace the material that was lost during the initial build. This action was repeated 12 times. Four different types of specimens were fabricated (some specimens with a section diameter of 2 mm, other specimens with a section diameter of 5 mm, and new powder and recycled powder for both dimensions). The specimens were referred to as small diameter (2 mm) and new powder (SN), small diameter (2 mm) and recycled powder (SR), large diameter (5 mm) and new powder (LN), and large diameter (5 mm) and recycled powder (LR). For specimens with a section diameter of 5 mm, the dimensions of the tensile specimens were set according to the ISO 6892-1 standard [23] (Figure 1). For specimens with a section diameter of 2 mm, we adopted the dimensions used in Persenot et al. [24,25] (Figure 1). Following the recommendations of the manufacturer, all specimens were subjected to a heat treatment to relieve stresses. The heat treatment was carried out in a protective atmosphere of Argon gas, in an oven chamber, with a ramp-up to 350 °C. This temperature was held for 45 min, and then we applied a new ramp-up to 850 °C for 60 min. Finally, the oven was cooled down to 100 °C.

Special specimens (5-mm-diameter spheres along the length of the principal stick) were designed to analyze the oxygen content of the interstitial gas in the as-built samples. They were built in the same fabrication batches as those used for the tensile and fatigue specimens. Oxygen content detection was performed by an inert gas fusion method via an ELTRA ONH 2000 elemental analyzer following the ASTM E1409-13 standard [26]. Six specimens were tested for each powder condition.

The arithmetical mean roughness (Ra), the total height of the roughness profile (Rt), and the mean roughness depth (Rz) were obtained according to the ISO 4287:1997 and ISO 4288:1996 standards [27,28]. Topographical data were acquired with an optical profiler (Sensofar-Tech SL, Plµ 2300, Barcelona, Spain) using a 20× EPI objective (637 × 477 μm). Data were taken from the center of the gauge length of the tensile probes. Six images were stitched together to obtain an extended measurement of the surface along the building direction of the probe. In order to obtain representative surface data, several extended images were recorded at equidistant zones (six in the case of the large specimens and four in the case of the small specimens). Images contained at least 97% of the measured points of the surface. Data processing, analysis, and visualization were performed using Gwyddion software (Gwyddion 2.59, open-source software, Brno, Czech Republic, http://gwyddion.net/, accessed on 31 July 2022). The surface data were filtered in order to correct the cylindrical form and to remove spurious points. Then, a high-pass filter was applied to assess the roughness parameters with a cut-off of 0.9 mm. Five profiles along the building direction were recorded in each extended topography image. Then, the Ra, Rt, and Rz parameters were estimated by averaging data among the corresponding profile set. Roughness parameters are given as the mean and standard deviation on measurements.

Quasi-static tensile tests were carried out at a strain rate of 0.001 s^−1^. A video extensometer was used to record the strain and strain-controlled tests were run. The tests were performed (on the four types of specimens) following the ASTM E8-E8M standard [29].

Fatigue tests were carried out following the ISO 1099:2017 standard [30]. Specimens with the same geometry as for the quasi-static tensile tests were used for fatigue tests. Three samples of each type of specimen were tested at a constant force ratio of R = 0.1 using a sinusoidal waveform at a frequency of 15 Hz. The maximum force in the fatigue tests was selected in order to apply a stress level of 300 MPa to the specimen. According to previous studies, with this stress level, the fatigue life is expected to be around 10^5^ cycles, which corresponds to a medium level of fatigue life for Ti-6Al-4V in PBF techniques [17,31]. The test was terminated when the sample was fractured. When the deviation from the mean number of cycles to failure was higher than 40% between specimens tested within any of the three samples, a fourth sample was tested.

After the fatigue tests, fracture planes of specimens were observed by scanning electron microscopy (SEM, JSM-6010LV, Tokyo, Japan). A fractographic analysis was carried out using an accelerating voltage of 5 kV and an emission current of 7000 nA at different magnifications. Fracture surfaces were examined to identify the defects responsible for crack initiation and subsequent propagation before the final fracture.

Unpaired *t*-tests were used to detect significant differences between new powder and recycled powder parameters of specimens with the same diameter. The significance level was set at an error probability of 5% (*p* < 0.05). The SPSS software (v. 22.0, IBM, Armonk, NY, USA) was used for the statistical analysis.

## 3. Results

### 3.1. Surface Roughness

**Figure 2:**
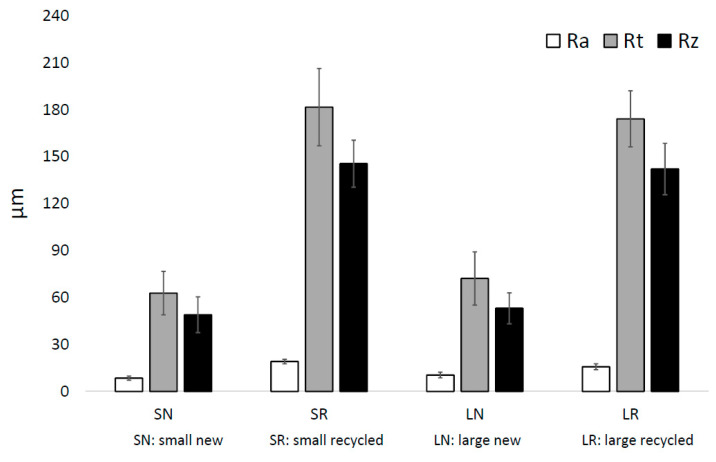
Mean and standard deviation of the arithmetical mean roughness (Ra), the total height of the roughness profile (Rt), and the mean roughness depth (Rz) of the LPBF Ti-6Al-4V specimens: small diameter and new powder (SN), small diameter and recycled powder (SR), large diameter and new powder (LN), and large diameter and recycled powder (LR).

**Figure 3:**
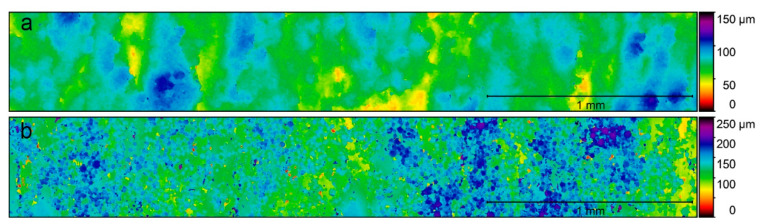
Surface topography in a false-color gradient of the small-diameter specimens fabricated with (**a**) new powder and (**b**) recycled powder.

**Figure 4:**
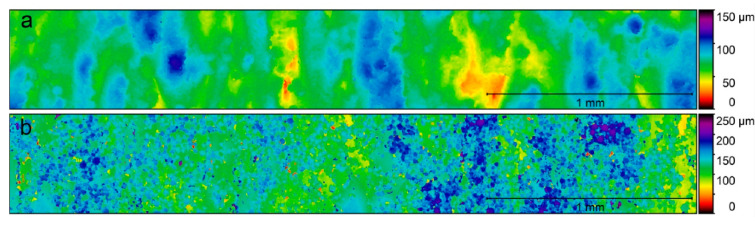
Surface topography in a false-color gradient of the large-diameter specimens fabricated with (**a**) new powder and (**b**) recycled powder.

For the analysis of the surface quality of the specimens, the Ra, Rz, and Rt were calculated from the topographical data obtained from the optical profiler. The mean and standard deviation values are shown in Figure 2. Significant differences (*p* < 0.05) between the Ra, Rt, and Rz values of the specimens fabricated with new powder and recycled powder were obtained, both for the small-diameter specimens and the large-diameter specimens. In the case of the small-diameter specimens, the Ra of the SR specimens was 225% higher than the Ra of the SN specimens, while in the case of the large-diameter specimens, this difference was 150%. Something similar happened with the Rz, which was 297% higher in the SR specimens than in the SN specimens and 268% higher in the LR specimens than in the LN specimens. In the same way, the Rt was 289% higher in the SR specimens than in the SN specimens and 241% higher in the LR specimens than in the LN specimens. Figure 3 and Figure 4 depict the conditioned surface topography of the samples colored in a false-color gradient.

There were no significant differences in the Rt and Rz values between small-diameter and large-diameter specimens of new powder. The same happened with the values of Rt and Rz between small-diameter and large-diameter specimens of recycled powder. In the case of Ra, there were significant differences. The Ra values of LN specimens were 124% higher than those of SN. However, the Ra values of SR specimens were 121% higher than those of LR specimens.

### 3.2. Mechanical Properties Obtained from Quasi-Static Tensile Tests and Oxygen Content

The values of the mechanical properties UTS, YS, E, and strain at failure (mean ± standard deviation) of all specimens obtained from the quasi-static tensile tests are shown in Table 1. No significant differences in the UTS and ST values between specimens fabricated with new powder and recycled powder were detected, both in the small-diameter and the large-diameter specimens. However, for both, there were differences between the strain at failure values, showing significantly lower values in the case of specimens fabricated with recycled powder. This is possibly related to the oxygen content. The specimens fabricated with recycled powder showed oxygen content values that were 119% higher than those fabricated with new powder.

**Figure 5:**
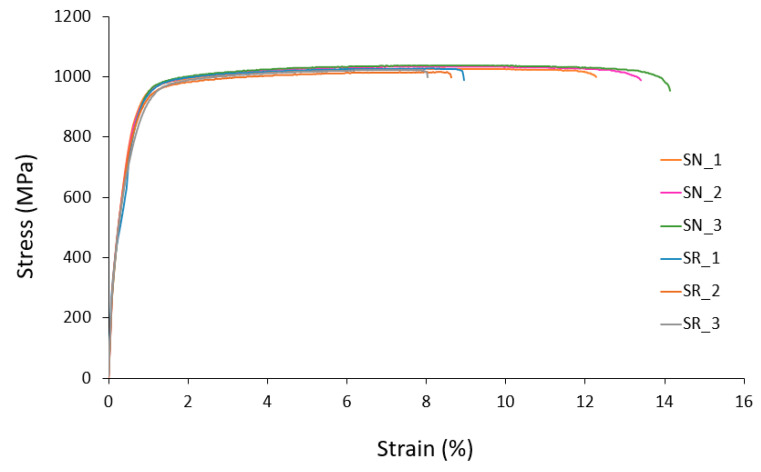
Comparison of tensile test results of 2-mm-diameter LPBF Ti-6Al-4V specimens fabricated with new and recycled powder.

**Figure 6:**
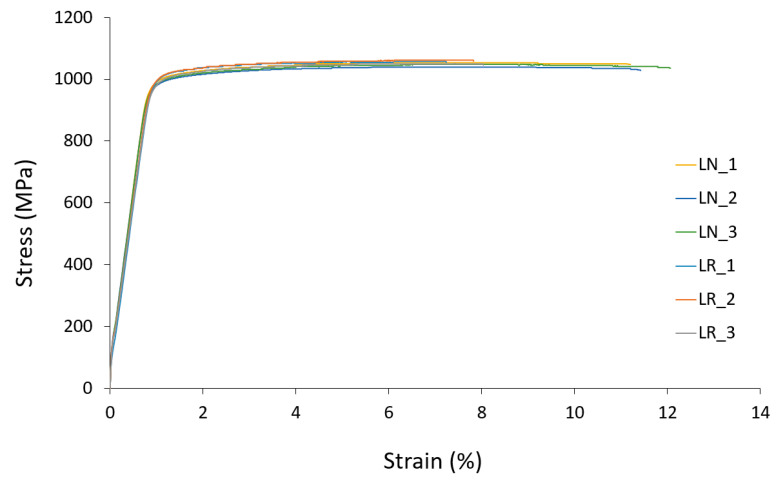
Comparison of tensile test results of 5-mm-diameter LPBF Ti-6Al-4V specimens fabricated with new and recycled powder.

The comparisons of stress–strain curves from the quasi-static tensile tests between the specimens fabricated with new and recycled Ti-6Al-4V powder, for both small-diameter and large-diameter specimens, are shown in Figure 5 and Figure 6, respectively.

### 3.3. Fatigue Behavior and Fractographic Analysis

**Figure 7:**
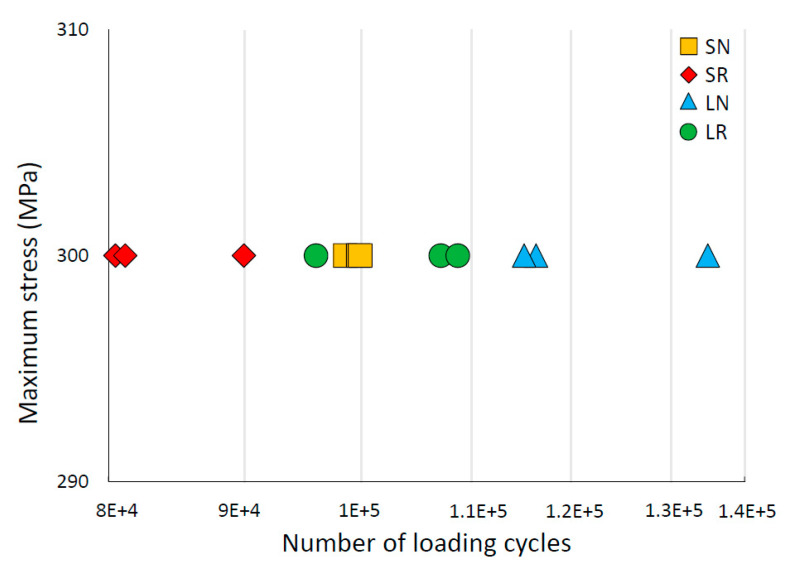
Fatigue results at the logarithmic scale of the LPBF Ti-6Al-4V specimens: small diameter and new powder (SN), small diameter and recycled powder (SR), large diameter and new powder (LN), and large diameter and recycled powder (LR).

The results of the fatigue tests performed on the specimens fabricated with new and recycled powder, for both dimensions, are shown in Figure 7. The specimens made of new powder demonstrated a longer fatigue life (expressed in terms of the number of cycles to breakage) than that of those made with recycled powder, both for the 2-mm-diameter and the 5-mm-diameter specimens. If the fatigue life is compared to the size of the specimens, it can be appreciated that the number of cycles to failure of the small-diameter specimens was slightly lower than that of the large-diameter specimens.

**Figure 8:**
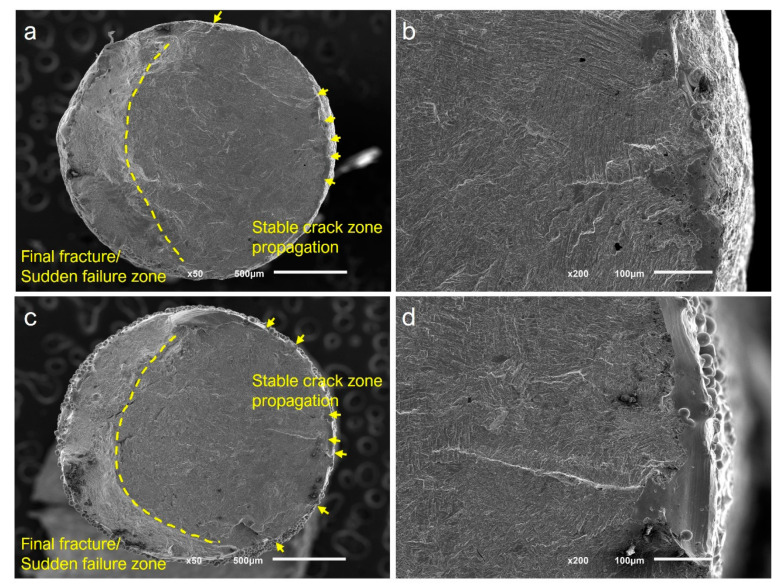
Representative SEM images of fracture surfaces after fatigue tests. (**a**,**b**) Samples fabricated with new powder; (**c**,**d**) samples fabricated with recycled powder. Multiple crack initiation sites at the surface (arrows) were observed for both powder conditions. The stable crack propagation zone and the final fracture zone are well delimited (dashed line).

Representative SEM images, obtained after fatigue tests, are shown in Figure 8. The fractographic analysis revealed that the fatigue crack initiation stage took place at the surface, where areas with defects such as pores, voids, or partially melted particles appeared. Secondary propagation cracks were observed, confirming the multiple locations of the origin of cracks at the surface. The perimeter defects where the crack initiation appeared (arrows), the area of fatigue crack growth with a smooth surface (stable crack zone propagation), and the ductile, sudden, and final failure area with a rough surface are shown in Figure 8a,c, corresponding to a small-diameter specimen fabricated with new and recycled powder, respectively. The internal defects played no role in the nucleation of cracks, as can be appreciated in Figure 8b,d.

**Figure 9:**
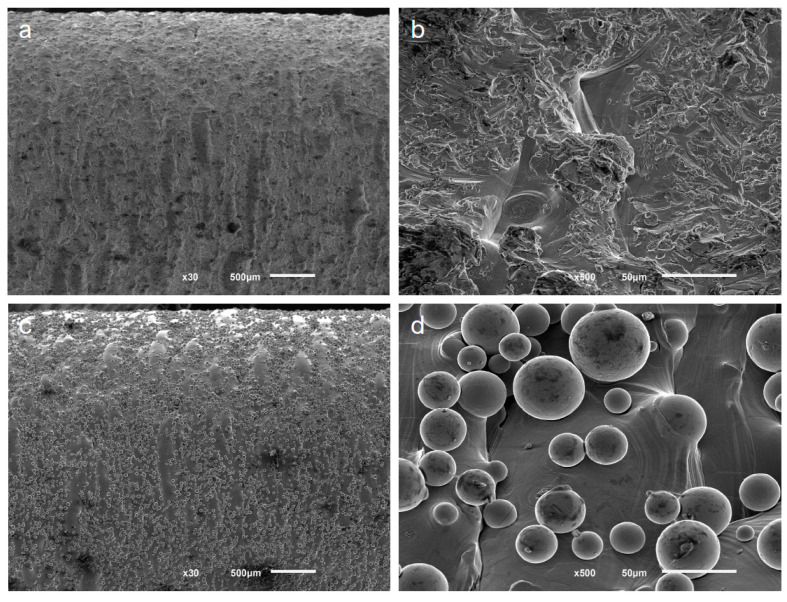
SEM images of the surface of small samples. New powder resulted in smoother surfaces and some defects can be observed (**a**,**b**). Recycled powder resulted in many partially melted powder particles adhered to the surface, creating a rough surface (**c**,**d**).

SEM images of the as-built surface of small-diameter samples are depicted in Figure 9. The features of the surface condition change with the recycling of the powder. The new powder produced a rough surface due to the presence of ripples. Those marks are left by the subsequent building layers, which are inherent to AM fabrication. Some other defects, such as voids and small sintered particles, can also be observed on the surface. The manufacturing process with recycled powder resulted in a similar rippled surface but many partially melted powder particles can be observed to be adhered to the surface, thus creating a rougher topography. The adhered particles had diameters in the range of 10–60 μm and retained a high degree of sphericity.

## 4. Discussion

In recent years, there has been a substantial increase in the application of medical implants obtained by additive manufacturing, especially by PBF technologies. One of the most used materials in this sector is the Ti-6Al-4V alloy, especially grade 23 or ELI. Due to the high cost of Ti-6Al-4V powders and the fact that only a small percentage of the raw material is used in each manufacturing batch, it is necessary to seek suitable powder reuse strategies. However, this recycling entails, in most cases, a change in the chemical and mechanical properties of both the titanium powder and the parts fabricated with this powder. Many authors have studied the change in features of Ti-6Al-4V powder, such as density, PSD, flowability, and microstructure, according to different recycling strategies. There is almost a total consensus on the conclusion that, with the recycling of Ti-6Al-4V powder in the LPBF technology, the density increases, the PSD remains relatively stable or tends to increase, the flowability improves, and the microstructure does not change [6,8,16,32]. However, there is no agreement in the literature regarding the mechanical properties of parts obtained by LPBF.

In this study, a large increase in the oxygen content was detected with the successive powder recycling cycles, despite the use of an inert gas in the manufacturing chamber. The specimens fabricated with recycled powder showed higher oxygen content values than those fabricated with new powder. This progressive increase in the oxygen content, with the successive recycling cycles of the Ti-6Al-4V powder in parts fabricated by PBF, was also evidenced in most of the previous studies [33,34]. However, the percentage increase in oxygen content depends on multiple factors, such as manufacturing parameters, heat treatments, and the recycling strategy used.

The increase in oxygen can cause changes in different mechanical properties of the fabricated parts [35]. In this study, no significant variations were observed in the values of the tensile strength (UTS and YS) with powder recycling. These values exceeded the minimum values recommended by the manufacturer and stated in standards ASTM F1472 and ASTM F3001 [36,37]. To this conclusion came also Carrion et al. [17], in whose study the changes in UTS and YS were negligible, despite having shown an increase in the oxygen content with the recycling of Ti-6Al-4V powder. However, other studies showed a slight increase in the tensile strength (UTS and YS) after build/reuse cycles [16,21,38]. On the other hand, a decrease in the UTS with powder recycling, due to the formation of larger pores near the surface of specimens, was shown by Seyda et al. [32]. In any case, the different strategies for recycling Ti-6Al-4V powder in the fabrication of parts with LPBF did not imply significant changes in the final values of the tensile strength. With this technology and material, powder recycling would probably have less of an influence on the final values of the tensile strength than the effect of different stress-relieving heat treatments [39].

In the literature, there is almost a total consensus on the need to emphasize the decrease in the elongation and ductility with the successive recycling cycles of Ti-6Al-4V powder of parts fabricated by LPBF [11]. This is mainly due to the increase in the oxygen content with the powder recycling cycles. However, Skalaton et al. [22] found no changes in the strain at failure of the specimens fabricated with reused powder, but a significant decrease in the Charpy impact strength was observed in samples produced from reused powder. In the present study, significant changes in strain at failure values were observed with the recycling of Ti-6Al-4V powder. In the case of the small-diameter specimens, the mean decrease in the strain at failure was 65%, while in the large-diameter specimens it was 66%. Slightly higher strain at failure values were obtained in the small-diameter specimens in comparison with the large-diameter ones. In the specimens fabricated with recycled powder, both in the small-diameter and the large-diameter specimens, the strain at failure values were moderately lower when compared with those obtained in another recycling study of Ti-6Al-4V specimens fabricated by LPBF [17]. This may be due to the type of heat treatment or the manufacturing parameters used. Annealing temperatures and times greatly influenced the strain at failure [40]. Yu et al. [41] reported values in PBLF Ti-6Al-4V pieces lower than those obtained in this study. This could be attributed, among other reasons, to the fact that lower temperatures were used in the heat treatment. After fabrication, parts were detached from the build plate, annealed at 900 °C for 1 h, and then furnace-cooled in an Argon environment. This heat treatment of PBFL Ti-6Al-4V has been reported to decompose its martensitic microstructure and result in lamellar α + β and a slightly higher fatigue strength as compared with stress relief at 740 °C for about 2 h [42].

In the PBF technology, large thermal gradients are produced in the material during the solidification of the successive layers that shape the parts due to the high focalization of the heat source and the short interaction times. In addition, together with the multilayer strategy, the partial fusion of powder particles and the stair-stepping effect caused by the geometry of the object induce a high degree of roughness on the surface of the fabricated parts [43,44]. Although this roughness can be beneficial in some biomedical applications—such as in bone scaffolds, where it is important to promote cell growth, as well as in the osseointegration of metallic implants—this is generally detrimental to mechanical properties. This is even more true for fatigue behavior, since roughness causes crack-initiating sites [19,21]. All these properties vary depending on the PBF manufacturing parameters and conditions [45,46].

In addition, temperature gradients depend on the surrounding thermal mass (the amount of material), which implies that the geometry of the piece influences the microstructure and roughness of the final parts. It has been reported that the microstructure of parts with small dimensions can change, and their roughness and internal pores become more critical due to the small cross-sectional area of the piece concerning the dimensions of the defect. This impoverishes their mechanical properties [14,47]. In this study, the question of how thermal gradients and recycled powder influence the roughness and the mechanical properties (especially the fatigue behavior) of specimens of different dimensions (i.e., small (2 mm diameter) and large (5 mm diameter)) has been considered. The first ones were chosen because the struts that make up the scaffolds used for large bone defects usually have small diameters. In these types of medical applications, mechanical loads play an important role. The large ones were chosen because they have dimensions that are widely used in these types of studies.

Ti-6Al-4V parts fabricated by LPBF usually exhibit a high degree of roughness (the average surface roughness ranges from 5 to 20 µm) [48,49]. These surface roughness values can be reduced if polishing techniques are employed, for example, with values between 0.3 and 0.6 μm in mechanical polishing, <1 μm in chemical polishing, and <10 μm in electrolytic polishing [49,50]. The roughness results show a significant increase in roughness with powder recycling. This increment is due to the variation in the surface topography resulting from the fabrication with recycled powder, as was observed by SEM. The surface of the samples fabricated with new powder was relatively smooth, as can be observed, because the manufacturing layers can be inferred (from the solidification of the melted layer). In contrast, the recycling of powder generated a rougher surface due to the large number of partially melted powder particles that adhered to the surface and the stair effect caused by the layer-by-layer fabrication notches. This increase in roughness was also observed in other studies of Ti-6Al-4V powder recycling with LPBF [17,32]. Regarding the influence of the size of the specimens, no significant difference was observed between the roughness of specimens of different diameters and the same powder, except for the Ra between large-diameter and small-diameter specimens, in which small differences were found, both in specimens with new and recycled powder. Despite the significant increase in roughness values with powder recycling, the Ra values remained well below those obtained in parts fabricated by EBM [19,25,50,51]. This implies a disadvantage from the biological point of view (the promotion of less cell adhesion), but it might entail an advantage in fatigue behavior.

In medical applications, cyclic loads (during walking, breathing, jaw clenching, etc.) play a fundamental role in the success of the implants. The fatigue results reveal that, for both dimensions, the fatigue strength of specimens fabricated with recycled powder was lower than that of specimens fabricated with new powder. This conclusion is in agreement with that of other authors [9]. On the other hand, the large-diameter specimens showed slightly better fatigue behavior compared with the small-diameter specimens. In high cycle ranges, this difference could be even more noticeable [52]. This is justified by the fact that, in parts obtained by PBF, where the surface roughness is high, especially in struts where the ratio between strut diameter and roughness is quite small, the fatigue strength diminished [19,53]. The machining or polishing of parts would imply a substantial improvement in the fatigue behavior. Therefore, it would be interesting to apply some of the polishing techniques to medical implants obtained by AM that do not require cell adhesion [54]. However, in those applications where cell growth is essential, such as in scaffolds applied to large bone defects, we recommend that they be left in the as-built condition. In these cases, the negative effect of cyclic loads can be mitigated with the progressive increase in cell growth [55].

The fractographic analysis in all of the specimens revealed that the fatigue failure was dominated by surface roughness, where the cracks initiated from the micro-notches on the surface rather than from internal defects. Therefore, the reuse of powder tends to play a key role in those personalized medical implants with complex designs and small sections or scaffolds with small-diameter struts, leading to an increase in the surface roughness that could reduce the expected fatigue lifespan.

In this work, only one fatigue stress level was studied. In future works, it would be interesting to obtain the complete S–N curves and analyze the effect of titanium powder recycling on the complete spectrum of load cycles. In any case, for the mentioned stress level, the fatigue strength values were in the same range as those obtained by other authors in as-built Ti-6Al-4V parts fabricated by LPBF [56,57]. Other researchers showed lower fatigue strength values at the 300 MPa stress level [31]. One possible reason for this is the great influence of roughness on fatigue behavior, especially in small-diameter parts. Another limitation of this study was the low number of static tensile test samples, although the low standard deviation values shown in the mechanical properties made it unnecessary to carry out a greater number of tests. An XRD study [58] of the phases of the materials formed due to the different recycling strategies and their relation with the mechanical properties should be carried out in the future. Additionally, we plan to study new recycling strategies and intermediate states of recycled powder.

## 5. Conclusions

In this study, the roughness and mechanical properties (obtained from tensile static and fatigue tests) of parts fabricated by LPBF with new and recycled Ti-6Al-4V ELI powder and with two different dimensions (specimens with diameters of 2 mm and 5 mm) were investigated. Based on the experimental results and observations, our conclusions can be summarized as follows:

1. Powder recycling had significant effects on the oxygen content, showing a substantial increase in the parts fabricated with recycled powder;
2. A significant increase in the surface roughness in parts fabricated with recycled powder was observed. However, no differences were observed in the roughness of the different sizes of parts, both for the specimens fabricated with new powder and recycled powder;
3. Powder recycling did not have any significant effects on the tensile strength (UTS and YS) values of the LPBF Ti-6Al-4V specimens;
4. Differences in the strain at failure were observed between parts fabricated with new and recycled powder, both in small-diameter and large-diameter parts. This implied a decrease in the ductility with the recycling of powder;
5. The fatigue strength of specimens fabricated with recycled powder was lower than that of specimens fabricated with new powder. On the other hand, the large-diameter specimens showed slightly better fatigue behavior compared with the small-diameter specimens;
6. The fractographic analysis revealed that the fatigue failure was dominated by surface roughness, where the cracks initiated from the micro-notches on the surface rather than from internal defects.

## Figures and Tables

**Table 1:** Mechanical properties of the LPBF Ti-6Al-4V specimens, given as the mean ± standard deviation.

	Ultimate Tensile Strength (MPa)	Yield Strength (MPa)	Young’s Modulus (GPa)	Strain at Failure (%)
Small diameter and new powder (SN)	1032.9 ± 5.2	967.0 ± 7.4	96.7 ± 7.8	13.2 ± 0.9
Small diameter and recycled powder (SR)	1021.7 ± 3.9	965.4 ± 4.8	86.6 ± 4.1	8.52 ± 0.47
Large diameter and new powder (LN)	1047.0 ± 7.2	993.2 ± 5.4	110.4 ± 6.4	11.6 ± 0.5
Large diameter and recycled powder (LR)	1057.1 ± 5.4	1003.7 ± 3.4	107.8 ± 1.2	7.7 ± 0.4

## Data Availability

Data are only available on request due to private restrictions.
